# PPARα ligand, AVE8134, and cyclooxygenase inhibitor therapy synergistically suppress lung cancer growth and metastasis

**DOI:** 10.1186/s12885-019-6379-5

**Published:** 2019-12-02

**Authors:** Lujin Wu, Wei Wang, Meiyan Dai, Huihui Li, Chen Chen, Daowen Wang

**Affiliations:** 1 Division of Cardiology, Department of Internal Medicine, Tongji Hospital, Tongji Medical College, Huazhong University of Science and Technology, and Hubei Key Laboratory of Genetics and Molecular Mechanisms of Cardiological Disorders, Wuhan, 430030 China; 2 Hubei Key Laboratory of Genetics and Molecular Mechanism of Cardiological Disorders, Huazhong University of Science and Technology, Wuhan, China

**Keywords:** PPARα agonist, AVE8134, Lung cancer, Indomethacin, EETs, 11-HETE

## Abstract

**Background:**

Lung cancer (LC) is one of the leading causes of death worldwide, which highlights the urgent need for better therapies. Peroxisome proliferator-activated nuclear receptor alpha (PPARα), known as a key nuclear transcription factor involved in glucose and lipid metabolism, has been also implicated in endothelial proliferation and angiogenesis. However, the effects and potential mechanisms of the novel PPARα ligand, AVE8134, on LC growth and progression remain unclear.

**Methods:**

A subcutaneous tumour was established in mice by injecting TC-1 lung tumour cells (~ 1 × 10^6^ cells) into their shaved left flank. These mice were treated with three different PPARα ligands: AVE8134 (0.025% in drinking water), Wyeth-14,643 (0.025%), or Bezafibrate (0.3%). Tumour sizes and metastasis between treated and untreated mice were then compared by morphology and histology, and the metabolites of arachidonic acid (AA) were detected by liquid chromatography-tandem mass spectrometry (LC-MS/MS). Inhibition of either Cyp2c44 expression by genetic disruption or cyclooxygenase (COX) activity by indomethacin was used to test the mechanisms by which AVE8134 affects tumour growth.

**Results:**

The pharmacodynamics effects of AVE8134, Wyeth-14,643, and Bezafibrate on lipids control were similar. However, their effects on tumour suppression were different. Eicosanoid profile analysis showed that all PPARα ligands reduced the production of AA-derived epoxyeicosatrienoic acids (EETs) and increased the hydroxyl product, 11-hydroxyeicosatetraenoic acids (11-HETE). Moreover, increased 11-HETE promoted endothelial proliferation, angiogenesis, and subsequent tumour deterioration in a dose-dependent manner possibly via activating the AKT/extracellular signal-regulated kinase (ERK) pathway. The increased 11-HETE partly neutralized the benefits provided by the Cyp2c44-EETs system inhibited by PPARα ligands in tumour-bearing mice. AVE8134 treatment worsened the tumour phenotype in Cyp2c44 knockout mice, indicating that AVE8134 has contradictory effects on tumour growth. The COX inhibitor indomethacin strengthened the inhibitory actions of AVE8134 on tumour growth and metastasis by inhibiting the 11-HETE production in vivo and in vitro.

**Conclusion:**

In this study, we found that the degrees of inhibition on LC growth and metastasis by PPARα ligands depended on their bidirectional regulation on EETs and 11-HETE. Considering their safety and efficacy, the novel PPARα ligand, AVE8134, is a potentially ideal anti-angiogenesis drug for cancer treatment when jointly applied with the COX inhibitor indomethacin.

## Background

Lung carcinoma is the most common cancer worldwide, with 1.8 million newly diagnosed patients per year, and has a higher mortality than that of the next top three cancers combined (158,080 vs 115,760 deaths/year) [1, 2]. While some treatments, such as radiation and immunotherapy, have given hope to lung cancer patients over the past few decades [3], its 5-year survival rate remains very poor [4]. That said, anti-angiogenesis treatments and the eradication and functional inhibition of tumour-associated endothelial cells (ECs), have emerged as important cancer treatments [5, 6]. However, the outcomes of current anti-angiogenic therapies that primarily target vascular endothelial growth factor (VEGF) pathways depend on cancer types and stages, and often lead to the development of resistance, hypertension, proteinuria, or even death [7]. Thus, more effective and safer anti-angiogenic therapies require further investigation.

Arachidonic acid (AA) released from membrane phospholipids is converted into various bioactive lipid mediators, such as epoxyeicosatrienoic acids (EETs), prostaglandins (PGs), and hydroxyeicosatetraenoic acids (HETEs), by either cytochrome P450 (CYP) epoxygenases, cyclooxygenase (COX), or lipoxygenase (LOX) pathways [8, 9]. Among them, EETs are particularly powerful pro-angiogenic eicosanoids and are positively linked with cancer progression [10, 11]. Cyp2c44, the functional homologue of human Cyp2c9, is one of the main CYP epoxygenases for EET biosynthesis in endothelial cells [12, 13]. Disruption of the *Cyp2c44* gene, or downregulation of its expression, reduces endothelial proliferation and tubular morphogenesis in vitro and inhibits primary tumour growth in vivo [12, 13]. Taken together, the Cyp2c44-EETs axis may be a vital target for cancer treatment, including lung cancer.

Peroxisome proliferator-activated nuclear receptor alpha (PPARα) is a ligand-activated nuclear receptor that modulates the transcription of specific target genes implicated in lipid metabolism and energy homeostasis [14, 15]. The PPARα-mediated transcriptional regulation of the *Cyp2c44* gene has been clearly established in previous studies [12, 16]. Once activated, PPARα translocates into the nucleus, and then binds to the PPAR response element (PPRE) in the promotor of the *Cyp2c44* gene and reduces its expression, thereby indicating why PPARα agonists inhibit angiogenic activity and tumour vascularization [12, 13]. Unfortunately, application of traditional PPARα agonists were restricted due its insufficient efficacy and hepatotoxicity [17].

As previously reported, AVE8134 is a specific and high-affinity ligand for PPARα, and shares with Wyeth-14,643 its PPARα selectivity and ability to improve plasma lipid profiles in rodents [18, 19]. More importantly, AVE8134 has been used in humans and has shown to be well tolerated at doses between 10 and 20 mg/kg body weight per day in contrast with Wyeth [18, 19]. We assume that, as with Wyeth, AVE8134 downregulates Cyp2c44 expression in the host endothelium, causing a decrease in the production of pro-angiogenic eicosanoid EETs and the inhibition of tumour vascularization, growth, and metastasis. We are proposing to repurpose AVE8134 as a safe agent for the treatment of human cancers.

## Methods

### Reagents

The Lipofectamine 2000 reagent was obtained from Invitrogen (Life Technologies Corporation, Carlsbad, CA). The primers for Cyp2C9 siRNA, and their controls were purchased from RiboBio (Guangzhou, China). The PPARα ligand AVE8134, 2-Methyl-6-({3-[(2-phenyl-1,3-oxazol-4-yl)methoxy]propoxy}methyl) benzoic acid, were synthesized by Dr. John R. Falck and kindly offered by Jorge H. Capdevila from the Department of Medicine (Division of Nephrology), Vanderbilt University, Nashville, USA. Wyeth-14,643, Bezafibrate, the PPARα antagonist GW6471, and the COX inhibitor indomethacin were purchased from MedChemExpress (New Jersey, USA). 11-HETE and four kinds of EETs were purchased from Cayman Chemical (Ann Arbor, Michigan, USA). For the purchasing information on some of the other conventional reagents in our lab, please refer to our previous articles [15, 20, 21].

### Cell culture

TC-1 tumour cells (#341334), originating from lung epithelial cells from C57BL/6 mice, were purchased from BeNa culture Collection (Sunzhou, China) and grown in RPMI 1640 medium supplemented with 10% fetal bovine serum (FBS) (Gibco, Grand Island, USA), 100 U/mL streptomycin, and 100 U/mL penicillin [22]. B16F10 melanoma cells (#TCM36) were obtained from the Cell Bank at the Chinese Academy of Science (Shanghai, China) and were maintained in Dulbecco’s Modified Eagle Medium (DMEM) with the aforementioned supplements [23]. Cells were grown in a humidified atmosphere of 95% air and 5% CO_2_ at 37 °C and harvested by 0.25% trypsin containing EDTA. The harvested cells were then used for tumour implantation. Human umbilical vein endothelial cells (HUVECs) (#CRL-1730) were obtained from American Type Tissue Collection (ATCC) and cultured in RPMI-1640 supplemented with 10% FBS [20]. For protein analysis or cell proliferation, cells were plated in 6-well and 96-well plates and treated by different concentrations of AVE8134, 11-HETE, or GW6471 for 48 h. Before plating for migration or angiogenesis experiments, HUVECs were first treated with AVE8134 for 48 h or 11-HETE for 24 h.

### Animal experiments

Wild-type C57BL/6 mice (10–12 weeks old) used for in vivo experiments were purchased from the Model Animal Research Center of Nanjing University (Nanjing, China). *Cyp2c44*^*−/−*^ mice were kindly provided by Artiom Gruzdev, Ph.D. (NIH/NIEHS) [13] and bred in the specific pathogen-free animal centre of Tongji Medical College. All the animals were maintained under 12-h light/12-h dark photoperiods with free access to water and food [21]. Tumour-bearing mice were established by subcutaneously injecting 1 × 10^6^ TC-1 cells or B16F10 cells in 50 μl phosphate-buffered saline (PBS) containing 50 μl Matrigel (BD Pharmingen) into the shaved left flank as previously described [13, 16]. Mice were considered tumour bearing when tumours became palpable at about 7–10 days after the first injection. Subsequently, the mice were divided into different treatment groups and this was considered the day 0 time point. Tumour growth was measured with a caliper using the formula V = W^2^Lπ/6, where V is the mean tumour volume, W is the mean short diameter, and L is the mean long diameter, and mice were humanely euthanized with an intraperitoneal injection of pentobarbital sodium (150 mg/kg) when tumours exceeded 20 mm in “L” diameter [23].

#### Experiment 1

TC-1 tumour-bearing mice were randomly divided into four groups according to the initial tumour sizes: (i) The equivalent concentration of dimethylsulfoxide (DMSO) was dissolved in drinking water as a control; (ii) 0.025% (g/ml) AVE8134 in the drinking water [19]; (iii) 0.025% (g/ml) Wyeth-14,643 in the drinking water [13]; (iv) 0.3% (g/ml) Bezafibrate in the drinking water [13]. Before being adjusted to the designated concentration in the drinking water, all PPARα ligands were solubilized by DMSO. The drinking water was changed every week.

#### Experiment 2

TC-1 or B16F10 tumour-bearing mice were treated with or without 11-HETE at a rate of 15 μg/kg/day by an osmotic mini-pump as previously described [21].

#### Experiment 3

*Cyp2c44*^*−/−*^ mice and littermate controls were induced into tumour-bearing, and then treated with or without 0.025% (g/ml) AVE8134 [19].

#### Experiment 4

TC-1 or B16F10 tumour-bearing mice were randomly divided into four groups: (i) the equivalent concentration of DMSO was dissolved in drinking water as a control; (ii) 0.01% (g/ml) indomethacin (COX inhibitor) alone in the drinking water [24]; (iii) 0.025% (g/ml) AVE8134 alone in the drinking water; (iv) 0.01% indomethacin and AVE8134 in the drinking water.

### Gene silencing

HUVEC cells were transfected with either siRNA against human *Cyp2c9* (100 nM) or a negative control (100 nM) siRNA using the Lipofectamine 2000 reagent according to the manufacturer’s protocol [21]. The medium was changed 4 h later and the cells continued to be cultured for an additional 48 h.

### Cells proliferation and migration

Endothelial cell proliferation was measured by bromodeoxyuridine (BrdU) incorporation assay using a BrdU Cell Proliferation Elisa Kit (Exalpha Biologicals, Inc., Massachusetts, CA, USA), according to the manufacturer’s instructions. In detail, HUVEC cells were cultured in 96-well plates at a density of 1 × 10^4^ cells/well in the presence or absence of AVE8134, EETs, or 11-HETE. Subsequently, the 1× BrdU Reagent was added to the wells for the final 2 h. Cells were fixed and permeabilized with Fixative/Denaturing Solution, and then incubated with an anti-BrdU detector antibody provided in the kit. The coloured reaction product was quantified using a spectrophotometer at a wavelength of 450 nm. Eight micrometre Boyden chambers (CostarCorning, New York, USA) were used to detect cell migration according to our established assay [23, 25]. Briefly, pretreated or transfected HUVEC cells (1 × 10^5^) were placed in the upper chamber with serum-free medium, while the lower chamber was filled with 200 μl of complete medium. The cells were allowed to migrate at 37 °C for 8 h, after which the non-traversed cells in the upper side were removed by cotton swabs, and the cells in the lower side were fixed with 4% paraformaldehyde and stained with crystal violet (Beyotime, Nantong, China) for 30 min. The stained cells were then counted under an optical microscope (Nikon, Tokyo, Japan).

### In vitro angiogenesis assay

For the Matrigel-based tubulogenesis assay, capillary-like structure formation was analysed as previously described [16]. Briefly, 96-well plates were pre-coated with 50 μl Matrigel (BD Biosciences, San Jose, CA, USA) for 30 min at 37 °C. After being stimulated by AVE8134 for 48 h or 11-HETE for 24 h, 1 × 10^5^ HUVEC cells were seeded into each well and cultured for another 4 h. The formation of capillary-like structures was then photographed, and the number of capillary-like structures was counted. The tube length was analysed by the AxioVision Rel software version 4.8 (Carl Zeiss AG, Jena, Germany).

### Liquid chromatography-tandem mass spectrometry (LC-MS/MS)

The metabolites in the cell lysates and tissues from mice were measured by LC-MS/MS (ACQUITY UPLC) in the laboratory of Prof. Yi Zhu at Tianjin Medical University as they previously described [26]. Briefly, 100 mg of tumour tissues, livers, or lungs were homogenized before lipid extraction. HUVECs were lysed by repeated freeze–thawing cycles and then pre-processed in methanol. After centrifugation, the supernatant was extracted by ethyl acetate twice, and then the upper organic phase was evaporated. The residue was then dissolved in 100 μl 30% acetonitrile. The resulting sample was then subjected to ultra-high-performance liquid chromatography (Waters, Milford, MA) with a 5500 QTRAP hybrid triple-quadruple linear ion trap mass spectrometer (AB Sciex, Foster City, USA) equipped with a Turbo Ion Spray electrospray ionization source.

### Western blot and quantitative real-time (qRT)-PCR

Proteins within tumour tissues and HUVEC cells were extracted using a Boster Kit (Bosterbio, CA, USA) according to the manufacturer’s instructions. Western blotting was performed as previously described [20]. The antibody, which was raised against the Cyp2c44’s IGRHQPPSMKDKMKC peptide (GenScript), was generated according to previous studies [13, 16]. Other antibodies used in this study are as follows: Glyceraldehyde 3-phosphate dehydrogenase (GAPDH) (Bosterbio), β-actin (Bosterbio), Cyp2c9 (Abcam, Cambridge, UK), COX1 (Santa Cruz, CA, USA), COX2 (Santa Cruz), Phosphorylated extracellular signal-regulated kinase 1/2 (p-ERK1/2) (Santa Cruz), ERK1/2 (Santa Cruz), P-AKT (Abcam), AKT (Abcam), and CD31 (Abcam).

For qRT-PCR, RNA from tumours was extracted using TRIzol (Invitrogen, Carlsbad, CA, USA) according to the manufacturer’s instructions and reverse-transcribed using the M-MLV First-Strand cDNA Synthesis Kit (Invitrogen) [15]. The mRNA levels of target genes were quantified by qRT-PCR using Power SYBR Green PCR Master Mix (Invitrogen) with the primers listed in Additional file 1: Table S1. GAPDH served as an internal control and the results were analysed using the 2^-ΔΔCt^ method.

### Histology and Immunohistochemical staining

Tumours and lung tissues were fixed in 4% paraformaldehyde and processed for histology or immunohistochemistry. For vascular density assessment, an FITC-conjugated CD31 antibody was used to label vascular endothelial cells and the percentage of CD31-positive structures/microscopic field was evaluated by Scion Imaging Software (Frederick, MD) [13]. The numbers of metastatic tumours in the lungs were assessed by hematoxylin-eosin (HE) staining as previously described [13].

### Detection of plasma lipid content and alanine aminotransferase (ALT)

The plasma triglyceride (TG) content was determined by colorimetric assays according to the product manual of a TG assay kit (Jiancheng bio, Nanjing, China) [15]. Briefly, plasma samples were extracted by the addition of 2:1 chloroform/methanol. The dried organic phase was then re-suspended in 100% ethanol and analysed using an enzymatic colorimetric method (GPO-PAP reagent, Rohe Diagnostics). The level of serum ALT was detected by an ALT assay Kit (Jiancheng bio).

### COX activity assay

COX Activity was analysed by a COX Activity Assay Kit (Cayman chem, Ann Arbor, MI). To determine COX2 activity, SC-560 was added into the reaction wells to eliminate all COX-1 activity.

### Statistical analysis

Data are presented as means ± SEM. The numbers of repetitions and groups are listed in the figure legends. Statistical analysis was based on a one-way ANOVA with a Tukey’s post hoc test; *P* < 0.05 was considered statistically significant.

## Result

### The novel PPARα ligand AVE8134 affected tumour growth and metastasis differently as compared to Wyeth-14,643 and Bezafibrate

**Fig. 1:**
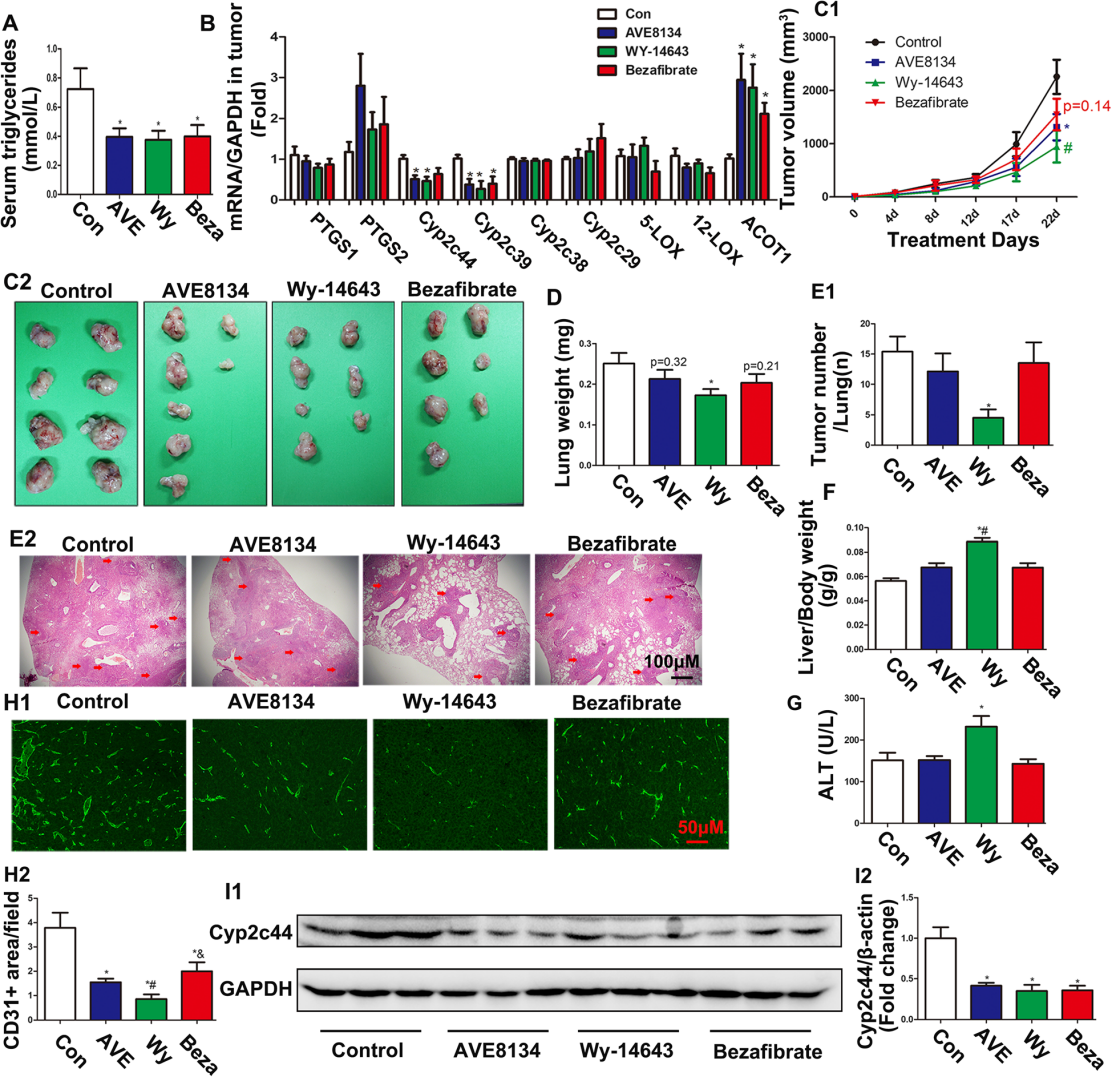
Different PPARα ligands exhibited different abilities to inhibit tumour sizes and metastasis. **a** Serum triglyceride levels in TC-1 tumour-bearing mice (*n* = 6–11). *P < 0.05, vs control. **b** Relative mRNA levels of *Ptgs1* (*Cox1*), *Ptgs2* (*Cox2*), *Cyp2c44*, *Cyp2c39*, *Cyp2c38*, *Cyp2c29*, Alox5, Alox12, *Acot1* (*n* = 6). *P < 0.05, vs control. **c1** and **c2** Images of the primary xenograft tumours and their growth curves in mice treated with three different PPARα ligands, AVE8134 (AVE), Wy-14,643 (Wy), and Bezafibrate (Beza; *n* = 8–11). ^*^P < 0.05, AVE vs control; ^#^*P* < 0.05, Wy vs control. **d** The weight of lungs in TC-1 tumour-bearing mice (*n* = 8–11). ^*^P < 0.05, vs control. **e1** and **e2** Hematoxylin and eosin (HE) staining and the number of lung metastatic tumours (red arrowhead; *n* = 8–11). ^*^P < 0.05, vs control. **f** The ratio of liver weight to body weight (*n* = 8–11). *P < 0.05, vs control; ^#^*P* < 0.05, vs AVE. **g** The levels of serum ALT (*n* = 6). *P < 0.05, vs control. **h1** and **h2** Tumour vascularization was quantified by CD31 antibodies in the paraffin sections of primary xenograft tumours. ^*^P < 0.05, vs control; ^#^*P* < 0.05 vs AVE; ^&^*P* < 0.05 vs Wy. (I1 and I2) Representative bands of Cyp2c44 and GAPDH in tumours were evaluated by western blot. ^*^P < 0.05, vs control (*n* ≥ 3)

AVE8134 is a new synthetic PPARα agonist that has excited many researchers given its anti-angiogenesis and anti-tumour properties, as well as its high PPARα affinity and low hepatotoxicity [18, 19]. However, whether it has any advantages in the treatment of cancer in comparison to other traditional PPARα agonists is still unknown. Thus, we compared AVE8134 with Wyeth-14,643 and Bezafibrate in TC-1 tumour-bearing mice. Although the concentration of plasma TGs and the expression of acyl-CoA thioesterase 1 (*Acot1)*, a downstream target gene of PPARα, were affected equally when mice were treated with the three PPARα agonists (Fig. 1a and b), these ligands showed different actions on tumour suppression and AA metabolism. As shown in Fig. 1c, after a 22 day treatment, AVE8134 inhibited TC-1 tumour sizes more than the untreated control group, but its effect were weaker than Wyeth-14,643 and stronger than Bezafibrate. As for metastasis, only Wyeth-14,643 significantly reduced pulmonary metastasis, as assessed by the weight of the lungs and HE staining (Fig. 1d and e). However, Wyeth-14,643 showed the strongest effects on hepatomegaly as compared to the other two PPARα agonists (Fig. 1f). AVE8134 failed to increase the level of serum ALT as Wyeth-14,643 did, indicating its advantages with respect to liver injury (Fig. 1g).

### PPARα agonists inhibited the expression of Cyp2c44 and the density of tumour vascular

Previous studies have reported that the levels of Cyp2c44 expression and EET biosynthesis, which are positively associated with tumour growth and angiogenesis, were controlled by PPARα activation in the endothelium [12, 13, 16]. The vessel density in tumours, as determined by anti-CD31 staining, was significantly inhibited by PPARα activation, with the strongest inhibition being observed for Wyeth-14,643, followed by AVE8134 and then Bezafibrate (Fig. 1h). Thus, we then detected the expression of four main Cyp2c subunits, Cyp2c29, 38, 39, and 44, in TC-1-bearing tumours. As shown in Fig. 1b and i, all three PPARα agonists decreased Cyp2c38 and Cyp2c44 expression in tumours.

### PPARα agonists altered the metabolites of AA

**Fig. 2:**
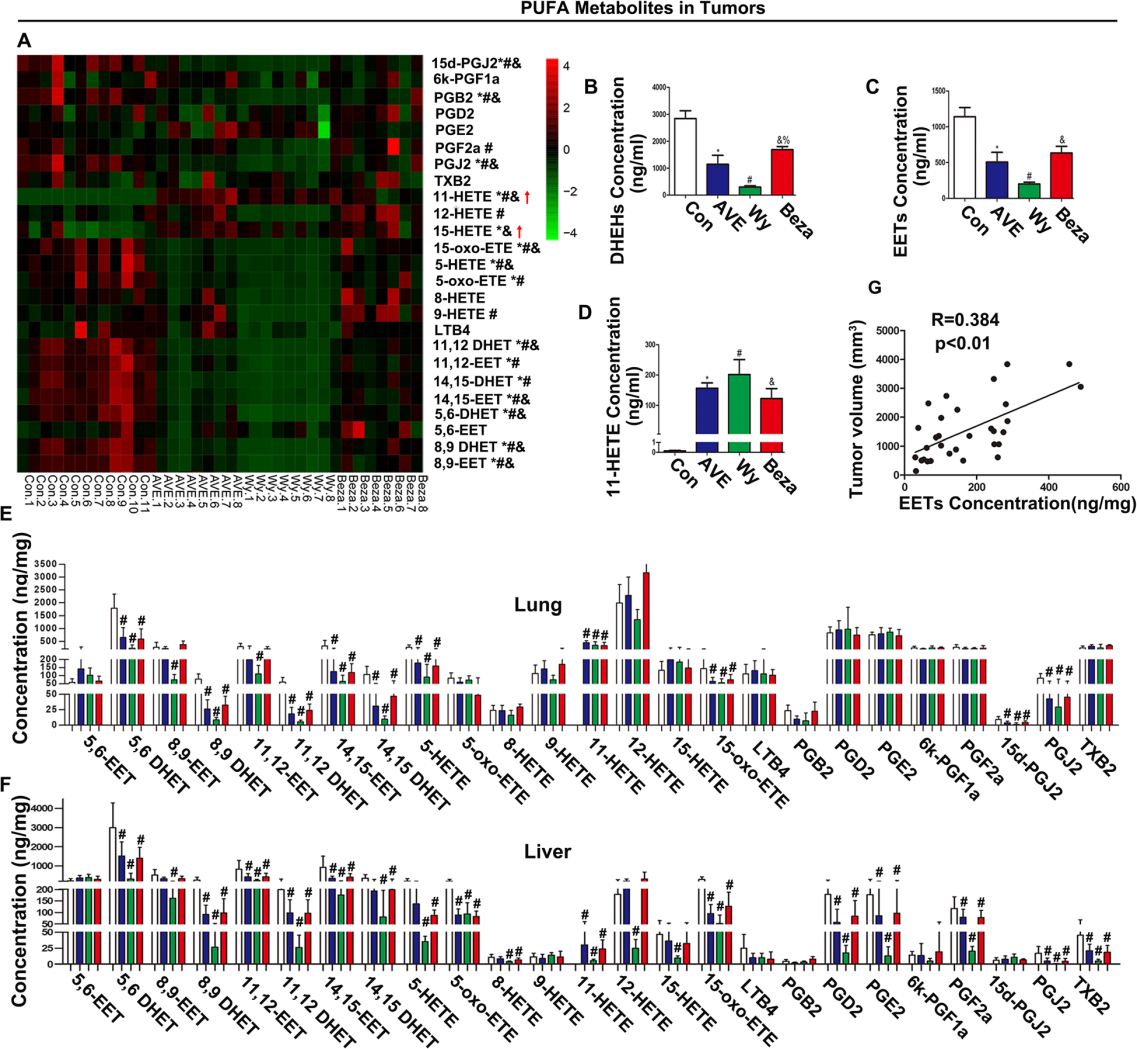
PPARα agonists altered the metabolites of AA in TC-1 tumour-bearing mice. **a** Heat map of 25 changed eicosanoids (derived from AA) in tumours (*n* = 8–11). ^* # &^*P* < 0.05, AVE, Wy, Beza vs control, respectively. **B** and **c** Levels of EETs and DHETs in tumours of PPARα agonist-treated mice (*n* = 8–11). ^* # &^*P* < 0.05, AVE, Wy, Beza vs control, respectively; ^%^*P* < 0.05, Beza vs Wy. **d** Levels of 11-HETE in tumours of PPARα agonist-treated mice (*n* = 8–11).^* # &^*P* < 0.05, AVE, Wy, Beza vs control. **e** and **f** Histograms showing eicosanoids profiles from lungs and livers of PPARα agonist-treated tumour-bearing mice (*n* = 8–11). ^* # &^*P* < 0.05 AVE, Wy, Beza vs control. **g** Correlation between EET concentration and tumour volume

Eicosanoids, including EETs, PGs, and HETEs, are a series of bioactive lipid molecules metabolized from arachidonic acid (AA) via three primary enzymatic pathways, COX, lipoxygenase (LOX) and cytochrome P450s (CYP), or in a nonenzymatic manner [8, 27]. AA-derived EETs are closely associated with tumour angiogenesis and development [11, 27]. Thus, we analysed 31 AA-derived metabolites in tumours, livers, and lungs from tumour-bearing mice by LC − MS/MS. Among them, six eicosanoids (including LXA4, 16-HETE, 17-HETE, 18-HETE, 19-HETE, 20-HETE) were undetectable in some samples and excluded. Changes in the other 25 eicosanoids are shown in the heat maps within Fig. 2a. Briefly, AVE8134 significantly reduced 13 eicosanoids (EETs, DHETs, PGB2, PGJ2, etc.), but increased two products (11-HETE and 15-HETE) in tumours (Fig. 2b–d). Most of these eicosanoids were also regulated by Wyeth-14,643 and Bezafibrate. Similar trends for most of these eicosanoids were observed in both lung and liver samples (Fig. 2e and f). Among them, EETs and 11-HETE were the most interesting, with EETs being a vital factor for tumour angiogenesis and 11-HETE being the only increased eicosanoid in all PPARα agonist-treated groups and tissues. AVE8134 showed weaker inhibition on EETs and DHETs than Wyeth-14,643 but stronger inhibition than Bezafibrate (Fig. 2b and c). As expected, the concentration of EETs in tumours was positively correlated with tumour size (Fig. 2g). The 11-HETE increased most in Wyeth-14,643-treated group, followed by AVE8134- and Bezafibrate-treated groups (Fig. 2d).

### AVE8134 inhibited endothelial proliferation, tube formation, and migration by activating PPARα

**Fig. 3:**
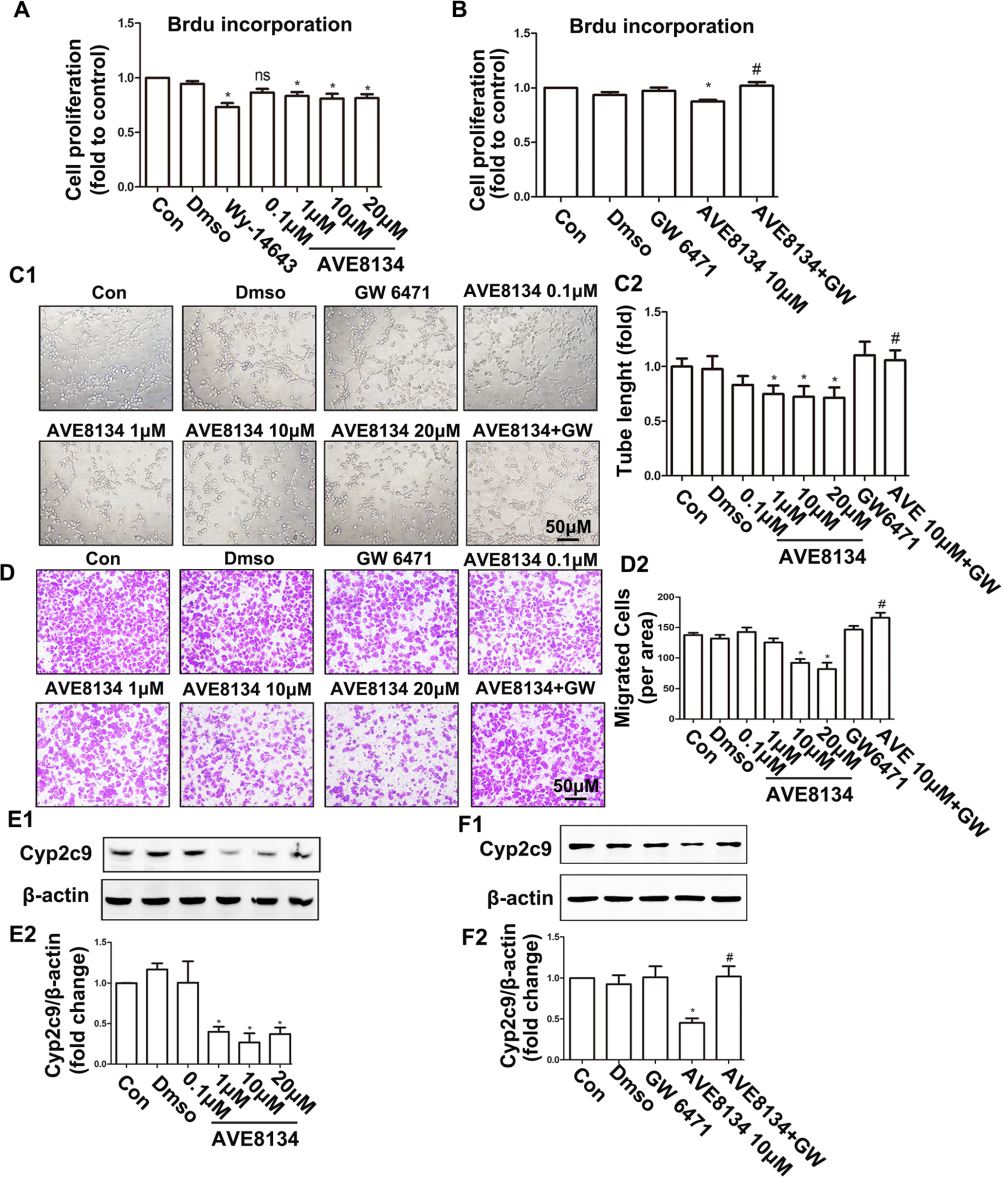
AVE8134 inhibited endothelial proliferation, tube formation, and migration by activating PPARα. **a** and **b** Proliferation ability of human umbilical vein endothelial cells (HUVECs) treated with Wy, different doses of AVE, or the PPARα antagonist GW6471, as assessed by BRDU incorporation (*n* = 5–7). ^*^P < 0.05 vs CON; ^#^*P* < 0.05 vs AVE8134 (10 μM). **c1** and **c2** HUVECs were plated into Matrigel after the indicated treatment. Shown are the representative images of capillary-like structures (*n* = 6). ^*^P < 0.05 vs CON; ^#^*P* < 0.05 vs AVE8134 (10 μM). **d1** and **d2** Treated endothelial cells were plated into Boyden chambers and cell migration was assessed (*n* = 8). ^*^P < 0.05 vs CON; ^#^*P* < 0.05 vs AVE8134 (10 μM). **e1**-**f2** Representative bands and the histogram of Cyp2c9/β-actin from HUVECs treated with Wy, different doses of AVE, or the PPARα antagonist GW6471 (*n* ≥ 3). ^*^P < 0.05, vs control; ^#^*P* < 0.05 vs AVE8134 (10 μM)

HUVECs were used to validate the effects of AVE8134 on angiogenesis in vitro. AVE8134 significantly inhibited endothelial cell proliferation, tube formation, and migration when the final concentration reached 1 μM (Fig. 3a–c), but its inhibitory action did not increase when the concentration increased. AVE8134’s inhibitory action was blocked by PPARα receptor antagonist GW6471 (Fig. 3b–d). Moreover, AVE8134 treatment significantly downregulated the expression of Cyp2c9 (the functional homologue of Cyp2c44 in mice) in HUVECs, which was also reversed by GW6471 (Fig. 3e and f).

### 11-HETE promoted endothelial proliferation and angiogenesis, causing tumour growth and lung metastasis

**Fig. 4:**
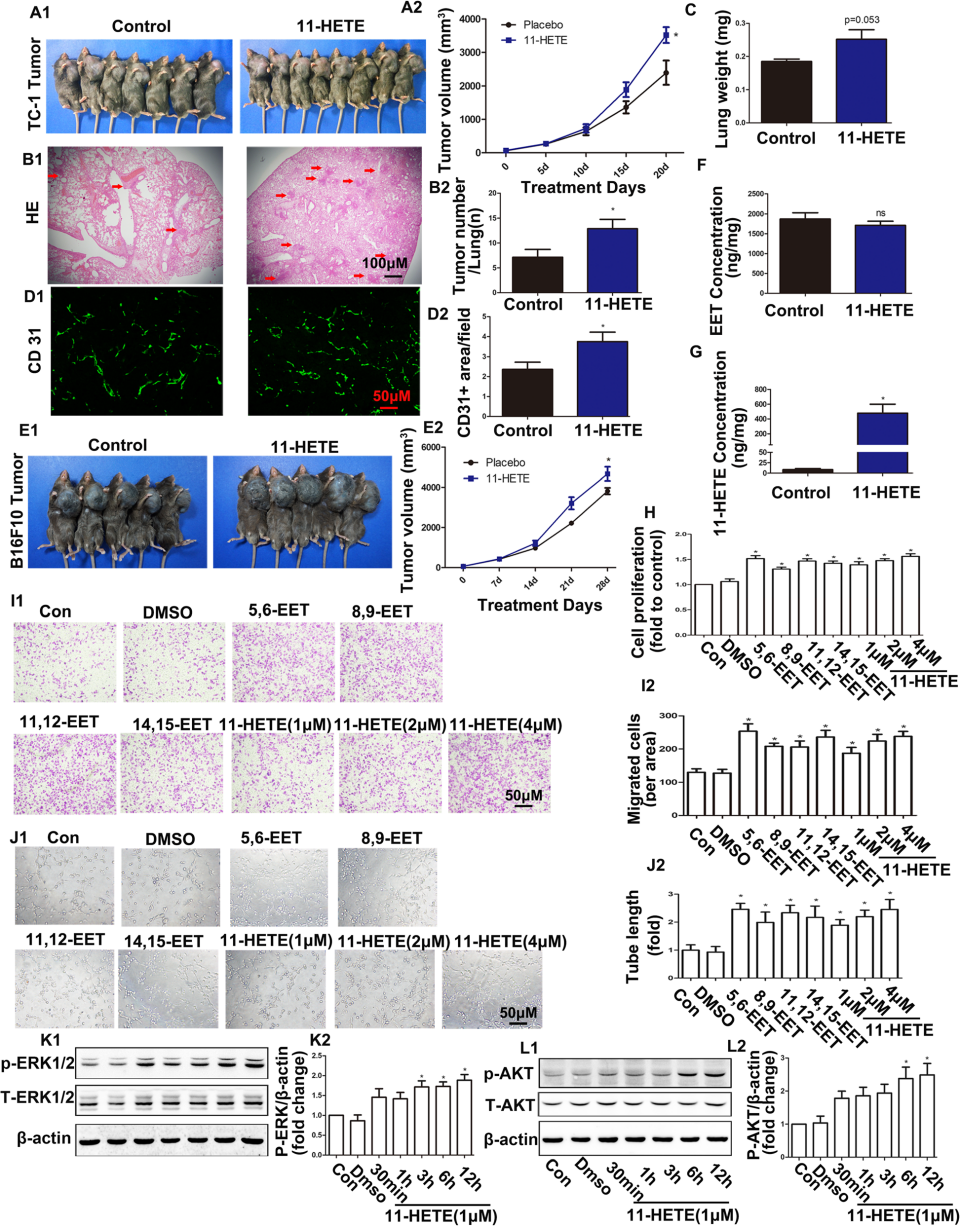
11-HETE promoted endothelial proliferation and angiogenesis, causing tumour growth and lung metastasis. **a1** and **a2** Images of TC-1 primary xenograft tumours and their growth curves in mice treated with or without 11-HETE (*n* = 8). ^*^P < 0.05 vs control. **b1** and **b2** Hematoxylin and eosin (HE) staining and the number of lung metastatic tumours (red arrowhead; *n* = 8). ^*^P < 0.05 vs control. **c** The weight of lungs in TC-1 tumour-bearing mice (*n* = 8). ^*^P < 0.05 vs control. **d1** and **d2** Tumour vascularization was quantified by CD31 antibodies in paraffin sections of TC-1 primary xenograft tumours (*n* = 8). ^*^P < 0.05 vs control. **e1** and **e2** Images of B16F10 primary xenograft tumours and their growth curves in mice treated with or without 11-HETE (*n* = 8). ^*^P < 0.05 vs control. **f** and **g** Levels of EETs and 11-HETE in TC-1 tumours of treated mice. ^*^P < 0.05 vs control. **h** Proliferative ability of HUVECs treated with EETs and different doses of 11-HETE, as assessed by BrdU incorporation (*n* = 4). ^*^P < 0.05 vs. control. **i1** and **i2** Representative images of migrated cells at the indicated treatment (*n* = 10). ^*^P < 0.05, vs control. **j1** and **j2** Representative images of capillary-like structures at the indicated treatment (*n* = 7). ^*^P < 0.05 vs control. **k1**–**l2** Western blot showing the activation of the AKT and ERK pathways under 11-HETE stimulation (n ≥ 3). *P < 0.05 vs control

We observed that 11-HETE was significantly increased in each PPARα treatment group, but its biological function was still unclear. In our in vivo experiments we found that increased 11-HETE significantly promoted the growth of TC-1 tumours (Fig. 4a). Moreover, lung metastasis and tumour angiogenesis in the 11-HETE treatment group significantly increased compared with the control group (Fig. 4b–d). In another tumour model, 11-HETE also promoted the growth of B16F10 melanoma cells (Fig. 4e). Thus, we concluded that 11-HETE promoted tumour growth, similar to EETs, and did not affect EET biosynthesis (Fig. 4f and g). In addition, we further revealed the effects of 11-HETE on endothelial function in vitro. As shown in Fig. 4h–j, 11-HETE promoted endothelial proliferation, cell migration, and tube formation in a dose-dependent manner. This may be related to its activation of the AKT/ERK1/2 proliferation signalling pathways (Fig. 4k and l).

### The increased 11-HETE partly counteracted the benefits of reduced EETs caused by PPARα activation on tumour treatment

**Fig. 5:**
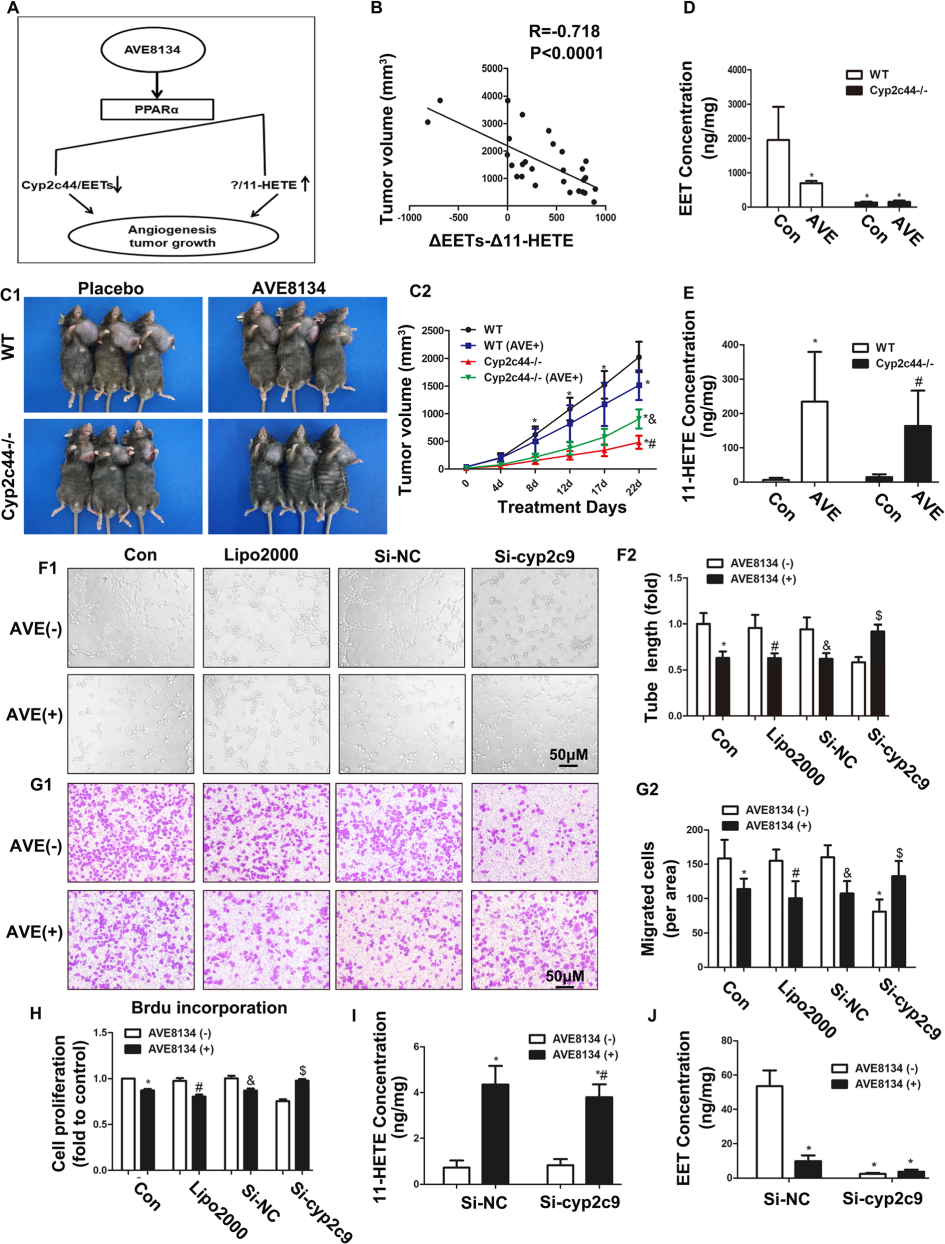
The increased 11-HETE counteracted the benefits from reduced EETs caused by PPARα activation on tumour treatment. **a** A schematic diagram showing the relationship between EETs and 11-HETE and how it is regulated by AVE8134. **b** ΔEETs and Δ11-HETE represents the difference between EETs and 11-HETE between PPARα ligand-treated groups and the control group. Shown is the correlation between ΔEETs - Δ11-HETE concentration and tumour volume. **c1** and **c2** Images of TC-1 primary xenograft tumours and their growth curves in WT and *Cyp2c44*^−/−^ mice treated with or without AVE8134 (*n* = 6–7). ^*^P < 0.05 vs WT placebo group; ^#^*P* < 0.05 vs WT AVE group; ^&^*P* < 0.05 vs *Cyp2c44*^−/−^ placebo group. **d** and **e** Levels of EETs and 11-HETE in TC-1 tumours (*n* = 6–7). ^*^P < 0.05 vs WT placebo group; ^#^*P* < 0.05 vs *Cyp2c44*^−/−^ placebo group. **f1**–**g2** Representative images of capillary-like structures and cell migration at the indicated treatment and their histograms (*n* = 7). ^*^P < 0.05 vs control; ^#^*P* < 0.05 vs lipo2000; ^&^*P* < 0.05 vs si-nc; ^$^*P* < 0.05 vs si-Cyp2c9. (H) Proliferative ability of HUVECs transfected with Cyp2c9 siRNA and then treated with or without AVE8134, as assessed by BrdU incorporation (*n* = 4). ^*^P < 0.05 vs control; ^#^*P* < 0.05 vs lipo2000; ^&^*P* < 0.05 vs si-nc; ^$^*P* < 0.05 vs si-Cyp2c9. **i** and **j** Levels of EETs and 11-HETE in HUVECs transfected Cyp2c9 siRNA and then treated with or without AVE8134 (*n* = 5). ^*^P < 0.05 vs si-nc; ^#^*P* < 0.05 vs si-Cyp2c9

As noted above, we found that PPARα reduced EET biosynthesis but increased the concentration of 11-HETE. Given that these are two opposite effects, we speculated that the inhibitory effect of PPARα agonists on tumour growth and angiogenesis depended on both EETs and 11-HETE levels (Fig. 5a). We used the value of ΔEETs - Δ11-HETE as a resultant vector and found that there was a negative linear correlation between ΔEETs - Δ11-HETE and tumour size (Fig. 5b). Subsequently, Cyp2c44 knockout (KO) mice were used to interdict the production of EETs. As shown in Fig. 5c–e, Cyp2c44 KO significantly reduced EET production but did not affect 11-HETE levels in TC-1 tumours, which led to a decrease in tumour size. Opposite to this effect in WT tumour-bearing mice, AVE8134 treatment increased tumour sizes in KO tumour-bearing mice compared with the KO controls (Fig. 5c). This may be because AVE8134 did not decrease the level of EETs in KO tumour-bearing mice, but increased the 11-HETE level instead (Fig. 5d and e). Our in vitro experiments showed that silencing Cyp2c9 alone or AVE8134 treatment alone significantly reduced the proliferation, migration, and tubular formation of endothelial cells (Fig. 5f–h). However, in the endothelium with low Cyp2c9 expression, the inhibitory effects of AVE8134 were reversed, which was closely associated with the increased production of 11-HETE (Fig. 5i and j).

### The COX1/2 inhibitor indomethacin enhanced the anti-tumour effects of AVE8134

**Fig. 6:**
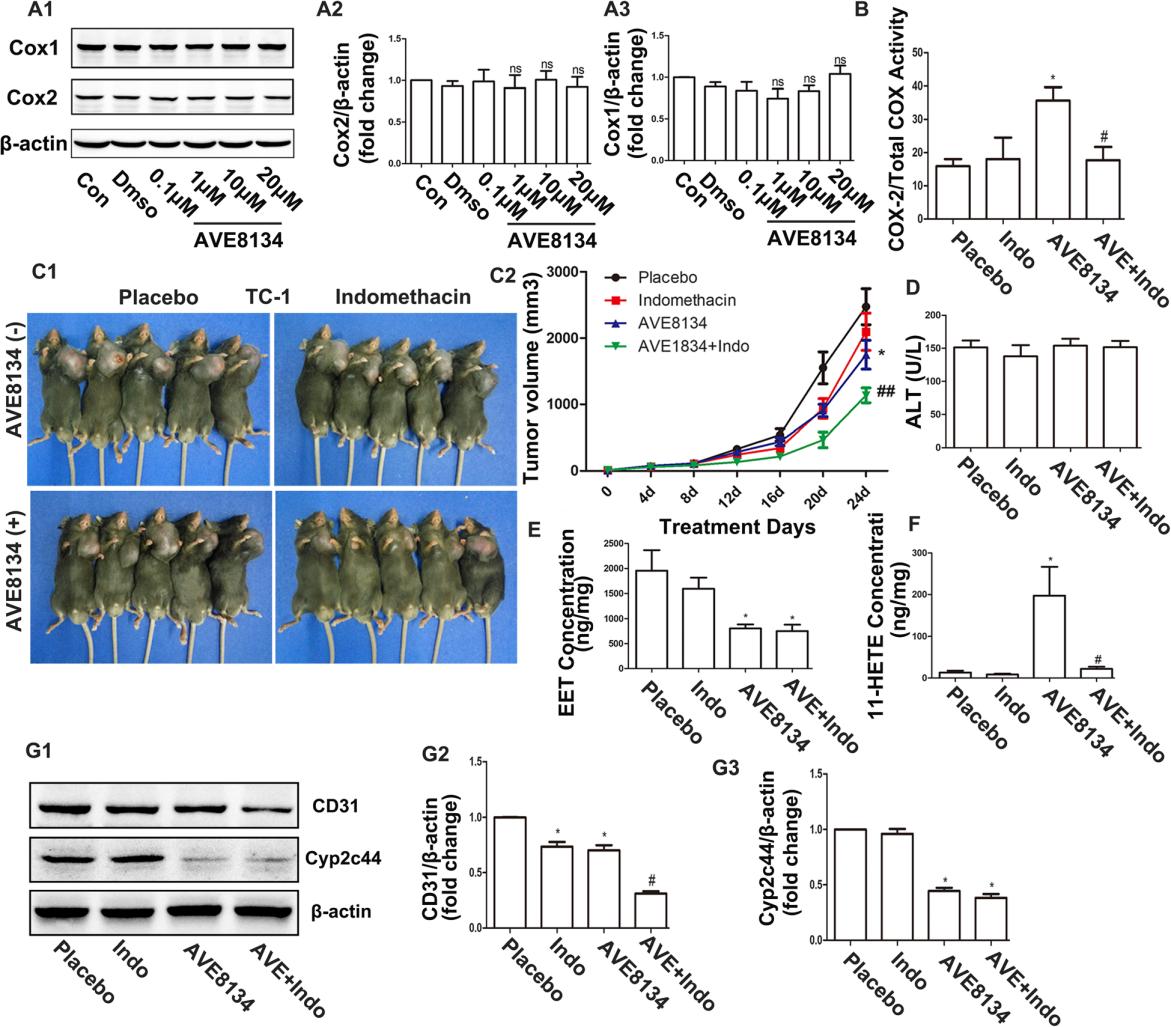
The cyclooxygenase (COX1/2) inhibitor indomethacin enhanced the anti-tumour effects of AVE8134. **a1**–**a3** Representative bands of COX1, COX2, and β-actin and the cartogram of COX1/β-actin and COX2/β-actin in HUVECs at the indicated treatment evaluated by western blot (n ≥ 3). ns = not significant. **b** The ratio of COX2 activity to total COX activity in TC-1 tumour lysates (*n* = 6). ^*^P < 0.05 vs control; ^#^*P* < 0.05 vs AVE. **c1** and **c2** Images of TC-1 primary xenograft tumours and their growth curves in mice treated with AVE, indomethacin, or both together (*n* = 8). ^*^P < 0.05 vs control; ^##^*P* < 0.01 vs AVE or indomethacin. **d** and **e** Levels of EETs and 11-HETE in HUVECs treated with AVE, indomethacin, or both together (*n* = 6). ^*^P < 0.05 vs control; ^#^*P* < 0.05 vs AVE. **f1**–**f3** Representative bands of CD31, Cyp2c44, and β-actin and the cartogram of CD31/β-actin and Cyp2c44/β-actin from TC-1 tumours at the indicated treatment, as evaluated by western blot. ^*^P < 0.05 vs control; ^#^*P* < 0.05 vs AVE (n ≥ 3)

**Fig. 7:**
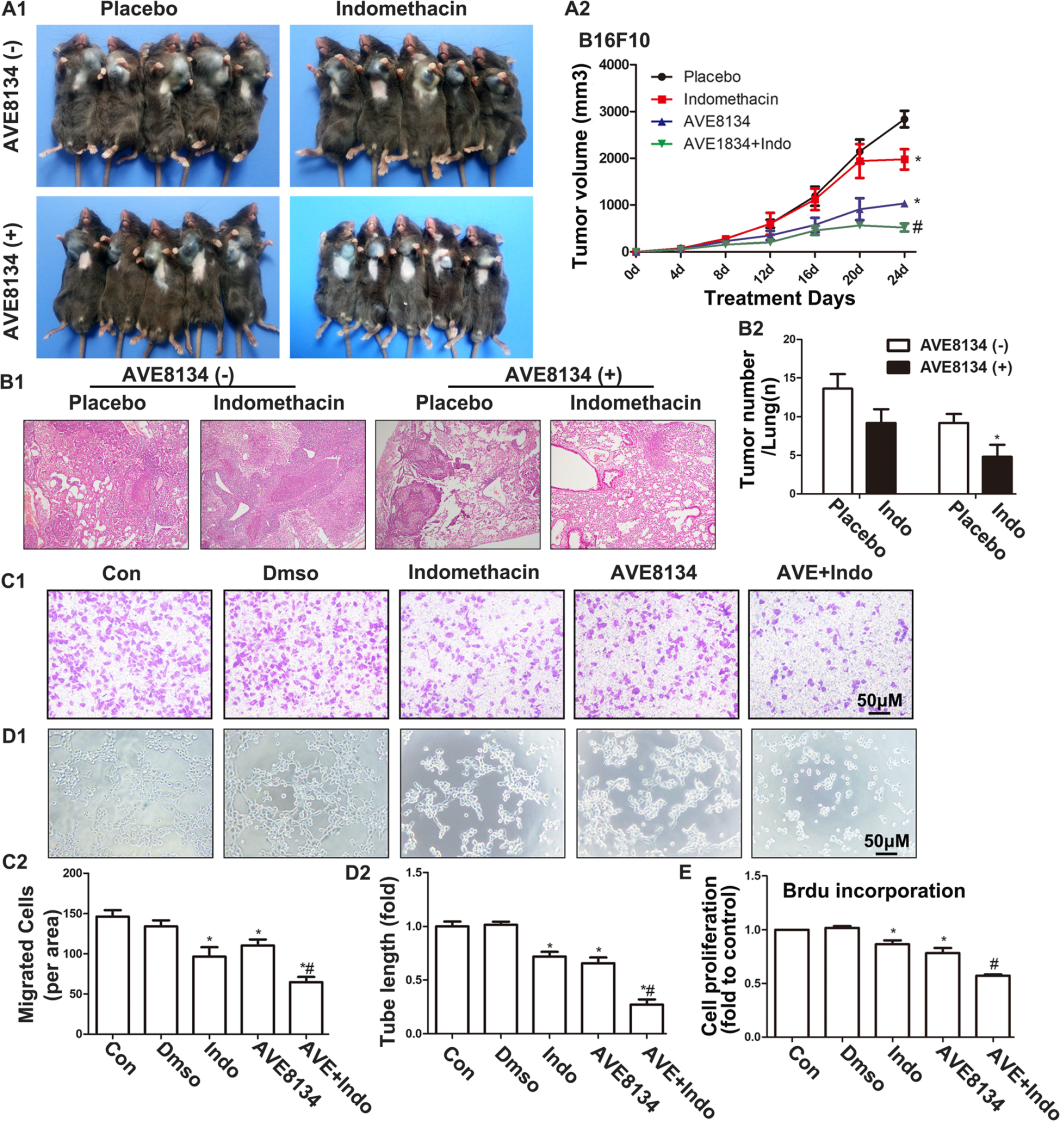
Indomethacin enhanced the anti-tumour effects of AVE8134 in the B16F10 model and in vitro. **a1** and **a2** Images of B16F10 primary xenograft tumours and their growth curves in mice treated with AVE, indomethacin, or both together (*n* = 8). ^*^P < 0.05 vs control; ^#^*P* < 0.05 vs AVE or indomethacin. **b1** and **b2** Hematoxylin and eosin (HE) staining and the number of lung metastatic tumours (*n* = 5). ^*^P < 0.05 vs placebo. **c1**–**d2** Representative images and their histograms of capillary-like structures and cell migration at the indicated treatment (*n* = 8). ^*^P < 0.05 vs control; ^#^*P* < 0.05 vs AVE. **e** Proliferative ability of HUVECs treated with AVE, indomethacin, or both together, as assessed by BrdU incorporation (*n* = 6). ^*^P < 0.05 vs control; ^#^*P* < 0.01 vs AVE

Although the main products of COX1 and COX2 are prostaglandins, these enzymes also convert AA to 11-HpETE, which is subsequently converted by the peroxidase activity to the corresponding 11-HETE [8, 28]. Previous studies have found that the COX inhibitor indomethacin reduced the formation of 11-HETE in bovine coronary artery endothelial cells [24, 29, 30] and that aspirin-acetylated COX was also responsible for the inhibition of 11-HETE production [31]. Although the expression of COX1 and COX2 was not changed in HUVECs, AVE8134 may have changed their catalytic structure (Fig. 6a). This was confirmed by the fact that AVE8134 shifted the COX1 activity to COX2 activity in tumours (Fig. 6b). Thus, we combined indomethacin with AVE8134 in an attempt to find a better therapeutic effect against tumours. Indomethacin failed to decrease the size of TC-1 tumours but significantly enhanced the inhibitory actions of AVE8134 (Fig. 6c) without causing liver damage (Fig. 6d). This may be because indomethacin blocked the increase in 11-HETE caused by AVE8134 (Fig. 6e and f). Moreover, the expression of CD31 was further inhibited by the addition of indomethacin as compared to the AVE8134 treatment alone (Fig. 6g). In the B16F10 melanoma model, indomethacin also synergistically inhibited tumour growth and and lung metastasis with AVE8134 (Fig. 7a-b). Similarly, AVE8134 treatment combined with indomethacin showed stronger inhibition of the proliferation, migration, and tubular formation of endothelial cells compared with either treatment alone (Fig. 7c–e).

In conclusion, we found that there were significantly differences in the effects of different PPARα ligands on tumour sizes and metastasis. Although AVE8134 was less beneficial with respect to tumour sizes and metastasis than Wyeth-14,643, it was much safer. Except for their inhibitory action on the Cyp2c44-EETs axis, the PPARα ligands also augmented the formation of 11-HETE. The increased 11-HETE promoted angiogenesis and tumour growth, counteracting the beneficial effects of the PPARα ligands. Indomethacin, a COX inhibitor, decreased 11-HETE, enhancing the anti-angiogenesis and anti-tumour effects of AVE8134. Thus, AVE8134 combined with indomethacin may have therapeutic potential against cancer, including lung cancer.

## Discussion

In this study, we proposed that AVE8134 acts as a novel anti-angiogenic drug that could be effective in the treatment of cancers. This is based on the fact that AVE8134 is a high affinity ligand for PPARα and is well tolerated in humans. Unfortunately, AVE8134 was not the strongest anti-tumorigenic and anti-angiogenic drug when compared with two other PPARα ligands and even failed to control lung metastasis. Metabolomics analysis found that PPARα ligands not only decreased EET biosynthesis by downregulating Cyp2c44 expression but also increased the production of a pro-angiogenic factor, 11-HETE, which in return counteracted their benefits on tumour suppression. That is, the effect of PPARα ligands on tumour inhibition depended on the variation in EETs and 11-HETE (ΔEETs-Δ11-HETE). Moreover, we found that the COX inhibitor indomethacin optimized the therapeutic effects of AVE8134 on tumour growth and angiogenesis via decreasing the formation of 11-HETE. Taken together, these results suggest that AVE8134 is an attractive drug for treating cancer if combined with indomethacin.

Angiogenesis is not only an important mechanism by which tumours obtain sufficient nutritional support and remove metabolic waste, but it is also required for the growth of numerous solid tumours and their metastases [32, 33]. Tumours remain in a dormant state until they become vascularized and these immature vessels increase the chance of tumour cells entering circulation and immigrating to distant organs [32, 34]. Angiogenesis is a complex multi-step process, involving endothelial cell proliferation, migration, sprouting, and transforming into tube-like structures [35]. A variety of endogenous factors, such as vascular endothelial growth factor (VEGF), basic fibroblast growth factors (bFGF), epidermal growth factor (EGF) and EETs, promote angiogenesis and contribute to the development of tumours. This makes these factors ideal targets for cancer treatments [36, 37]. CYP epoxygenases and their metabolites, EETs, are upregulated in human tumours and have been identified as powerful pro-angiogenetic mediators [23, 38]. Overexpression of CYP epoxygenases or inhibiting EET hydrolysis by soluble epoxide hydrolase inhibitors (sEHi) demonstrated their capacity to promote tumour growth and metastasis in many preclinical studies [23, 34]. CYP epoxygenase inhibitors will hopefully enter into clinical trials for cancer treatments [23, 39]. PPARα agonists exhibited a reduction in tumour growth and vascularization by suppressing Cyp2c44 expression, which connects them with clinical tumor treatment [12, 13, 16].

PPARs are members of the steroid receptor superfamily and there are three subtypes: PPAR-α, −δ, and -γ. These receptors are important ligand-activated transcription factors involved in the regulation of cell proliferation and energy metabolism [15, 40]. Interestingly, increasing evidence has showed that PPAR activation exhibited multiple functions in tumour progression [41]. Compared with the unified conclusion that PPARγ inhibited the growth of various tumours [42], the effects of PPARα on tumour progression were diverse and depended on the tissue type or PPARα ligands [13]. In some studies, PPARα deficiency inhibited tumorigenesis through increasing the endogenous angiogenesis inhibitor thrombospondin-1 (TSP-1) [43]. Moreover, PPARα activation enhances breast cancer cell proliferation by upregulating cyclin E levels [44]. Conversely, PPARα activation with Wyeth-14,643 or fenofibrate was also reported to inhibit endothelial cell growth and non-small cell lung cancer (NSCLC) progression via binding to the PPER area in the promoter of mouse *Cyp2c44* [12, 13, 16]. Besides the paradoxical roles of PPARα, another nonnegligible issue impeding its use in clinical trials is that PPARα agonists typically increase the incidence of liver hepatomegaly and tumours through induction of cell proliferation and oxidative stress [17]. This study compared the effects of three different PPARα agonists on tumour progression and liver hepatomegaly and found that the novel PPARα ligand AVE8134 was an ideal choice for tumour treatment given its effectiveness and safety. Inconsistent with previous studies, this study suggests that PPARα ligands not only reduce EET production via downregulating Cyp2c44 expression but also increase 11-HETE biosynthesis. Increased 11-HETE was shown to be a pro-angiogenic and -tumorigenic factor, which partially cancelled out the benefits from decreased EETs in tumour-bearing mice. Thus, the combined utilization of drugs that inhibit 11-HETE formation may solve this issue and enhance the anti-tumour effect of AVE8134.

11-HETE, a bioactive metabolite derived from AA, is mainly generated from COX enzymes, while LOX and CYP enzymes, and non-enzymatic catalytic pathways may also contributed to its production [30, 31]. Previous research has reported that the COX2 specific inhibitor, celecoxib, reduced 11-HETE production in lung cancer A549 cells [45]. AA can bind to the COX active site in a specific catalytic arrangement that leads to 11-HETE production, which is inhibited by aspirin treatment [29, 46]. Moreover, the non-specific COX inhibitor indomethacin was observed to reduce the formation of 11-HETE in bovine coronary artery endothelial cells [29, 30]. These results suggest that the formation of 11-HETE relies on COX enzymes. Although increased 11-HETE is described as a biomarker ranging from coronary events to cancers, its biological function remains unclear [47]. This study found that 11-HETE stimulated endothelial proliferation, migration, and angiogenesis, as well as the subsequent growth and metastasis of tumours.

Although the control region of the *COX2* gene possesses one response element for PPAR (PPRE), there is no evidence that indicates that PPARα activation affects its expression [48]. However, a previous study has reported that PPARγ activation participates in the transcriptional activation of the *COX2* gene [49]. That said, PPARα may change the catalytic arrangement at the COX active site. This may be important given the fact that COX-2 is an inducible isoform of COXs and its overexpression is linked to various cancers [50]. Both COX1 and COX2 inhibitors have been reported to inhibit tumour progression and this inhibition underlies their anti-inflammation and anti-angiogenesis effects [51, 52]. The COX inhibitor indomethacin has been used previously as a non-steroidal anti-inflammatory drug for the treatment of various inflammatory diseases, such as arthritis, fever, and various headache syndromes [53]. In subsequent preclinical and clinical studies, researchers found that indomethacin exhibits anti-tumour activity [52], although the underlying mechanisms are unclear. This study found that indomethacin synergistically strengthened the anti-tumour effects of AVE8134 by inhibiting the production of 11-HETE. Although indomethacin alone inhibited the activation of endothelial cells and slightly suppressed the growth of TC-1 lung tumours, its joint effects with AVE8134 seem more powerful. Thus, combining the novel PPARα ligand AVE8134 with the COX inhibitor indomethacin provides a new and effective strategy for the treatment of cancer.

## Conclusions

In this study, we revealed that PPARα ligands were able to change the levels of AA-derived metabolites. The novel PPARα agonist AVE8134 showed advantages at security in tumor treatment, although its anti-tumor effect was inferior to wyeth-14,643. Except for the well-known downregulation of CYP2c44 expression and subsequent EET synthesis, AVE8134 significantly increased the levels of 11-HETE, possibly through changing the catalytic activity of COX enzymes. Increased 11-HETE facilitates angiogenesis and tumour progression, which was effectively blocked by the COX inhibitor indomethacin. The combined treatment of indomethacin with AVE8134 may be an ideal and effective drug combination for the treatment of cancer, including lung cancer.

## Supplementary information

**Additional file 1.** Primers For qRT-PCR.

## Data Availability

The authors declare that materials described in the manuscript, including all relevant raw data, will be available from the corresponding author upon reasonable request.

## Abbreviations

11-HETE: 11-Hydroxyeicosatetraenoic acids
AA: Arachidonic acid
AVE8134: 2-Methyl-6-({3-[(2-phenyl-1,3-oxazol-4-yl)methoxy]propoxy}methyl) benzoic acid
bFGF: Basic fibroblast growth factors
BrdU: Bromodeoxyuridine
COX: Cyclooxygenase
CYP: Cytochrome P450
EETs: Epoxyeicosatrienoic acids
EGF: Epidermal growth factor
ERK: Extracellular signal-regulated kinase
HETEs: Hydroxyeicosatetraenoic acids
HUVECs: Human umbilical vein endothelial cells
LC: Lung cancer
LC-MS/MS: Liquid chromatography-tandem mass spectrometry
LOX: Lipoxygenase
NSCLC: Non-small-cell lung cancer
PGs: Prostaglandins
PPARs: Peroxisome proliferator-activated receptors
PPARα: Peroxisome proliferator-activated nuclear receptor alpha
PPRE: PPAR response element
sEHi: Soluble epoxide hydrolase inhibitors
VEGF: Vascular endothelial growth factor
