# Supplementary material for: PPARα ligand, AVE8134, and cyclooxygenase inhibitor therapy synergistically suppress lung cancer growth and metastasis
Source: BMC Cancer. 2019 Dec 2;19:1166. doi: 10.1186/s12885-019-6379-5 (PMC6889744; doi:10.1186/s12885-019-6379-5)
Supplement: Supplementary file 1 — Additional file 1. Primers For qRT-PCR. [file 12885_2019_6379_MOESM1_ESM.docx]

Primers For qRT-PCR.

| Gene name | Forward | Reverse |
| --- | --- | --- |
| m_PTGS1 | GTCCTTCTCCAATGTGAGCTAC | CAGGAATGAACTCCCTTCTCAG |
| m_PTGS2 | CTCACGAAGGAACTCAGCAC | GGATTGGAACAGCAAGGATTTG |
| m_Cyp2c29 | TCAGCCAATCCTTCACCAAC | CTTCCTCTACCAGCAAACTCC |
| m_Cyp2c38 | ATGGGTATGAAGCAGTGAAGG | TGAACCGTCTTGTCTCTTTCC |
| m_Cyp2c39 | CACTTGAAAATCTGGCCTGC | ACATGGTCAATCTCTTCCTGG |
| m_Cyp2c44 | GACCTCTGCCCATTATCGAAG | CTAGGCTTCTCTTCCCCATTC |
| m_Alox5 | TGTCTGAGGTGTTTGGTATCG | AAGGCCATACTCGCAGATAAG |
| m_Alox12 | CCGCATAGAGAACAGTATCACC | GTGGAGTGTTTAGTCAGGAGAG |
| m_Acot1 | ACTACGATGACCTCCCCAAG | CATAGCAAGGCCAAGTTCAC |
| m_GAPDH | CTTTGTCAAGCTCATTTCCTGG | TCTTGCTCAGTGTCCTTGC |
